# Tip efficiency of a customized lingual appliance: Performance of wires with two different ligatures

**DOI:** 10.1111/eos.13031

**Published:** 2024-12-16

**Authors:** Sara Drago, Alberto Lagazzo, Anna De Mari, Luigi Rizzi, Roberto Stradi, Maria Menini, Marco Migliorati

**Affiliations:** ^1^ Department of Orthodontics Genova University Genova Italy; ^2^ Section of Materials Engineering, Department of Civil, Chemical and Environmental Engineering (DICCA) Genova University Genova Italy; ^3^ Department of Orthodontics Pavia University Pavia Italy; ^4^ School of Orthodontics and Temporomandibular Disorders University of Naples Federico II Naples Italy; ^5^ Department of Surgical and Diagnostic Sciences Genova University Genova Italy

**Keywords:** brackets/wires, lingual orthodontics, mechanical properties, tip control

## Abstract

Tip control in lingual orthodontics may be challenging because of the presence of a vertical slot and the particular configuration of the customized appliances. The aim of this in vitro experimental study was to investigate the role of the ligature–wire–slot system in achieving better tip control. A set of customized lingual brackets was obtained for a dedicated typodont made of extracted human teeth. A compression/traction machine tested two types of ligatures in combination with seven different wires, and the tipping angle of each configuration was derived. A statistically significant difference was found between ligatures when the complete set of data was tested. In addition, differences between ligatures were found when testing each wire separately. A statistically significant difference was found among all wires. Full‐size wires showed the smallest angles, which correspond to the greatest efficiency of the slot–archwire–ligation system in terms of tip control, and this efficiency appeared to be ligature‐related. The role played by the type of ligature was more relevant for undersized wires.

## INTRODUCTION

Lingual orthodontic therapeutic modalities have been improved by customization and have become excellent alternatives for patients and clinicians who require an invisible treatment [1, 2, 3, 4]. Nowadays, a lingual orthodontic therapy can be used to treat every type of malocclusion that would usually be treated by means of the labial technique [5]. The orthodontic foundation is the opportunity to push the tooth into the correct three‐dimensional position. When each single tooth is in the proper position, all teeth are in the correct three‐dimensional spatial position. These principles allow orthodontists to reach the objectives set by Andrews, known as the six keys of occlusion [6]. Tooth‐position control is managed differently in lingual orthodontics than in labial orthodontics; in lingual orthodontics the point at which force is applied is located more apically than in labial orthodontics, and this implies different geometry and biomechanics, especially in the vertical plane, although the treatment phases are the same [7, 8, 9].

The three‐dimensional control of the position of teeth is always of fundamental importance. However, this is more difficult to achieve in the lingual technique than in the labial technique because of a reduction of the interbracket distance, the complex lingual surface anatomy, and the different materials used. The fundamental unit of fixed orthodontic mechanics is the wire–bracket system; this unit must allow full control in the three planes of space [10, 11].

To obtain tooth movement, proper biomechanics should be applied, and it is recommended for the wire–bracket system to be used in a range between 50 and 100 cN [12]. The application of wires with different moduli of elasticity and dimensions allows the clinician to place a bracket‐wire‐ligature system simultaneously with providing a workflow in which the position of each tooth and the coordination of the dental arches is finalized. The aim of this research was to investigate, through the use of different ligature‐wire‐bracket configurations, how the bracket–wire–ligature system may influence the three‐dimensional control of the tip. The null hypothesis was that there would be no difference among the different configurations.

## MATERIAL AND METHODS

### Dental model

In order to evaluate the properties of the single materials, a study was designed in which eight extracted human teeth were examined: one right upper central incisor, two upper lateral incisors (right and left), two upper canines (right and left), two first upper premolars (right and left), and a second upper premolar (left). Each individual tooth was carefully cleaned using manual tools and with the use of ultrasound, carefully disinfected using a hydrogen peroxide solution (3%), then stored in a physiological saline solution of 0.9% sodium chloride (pH 5.5) in order to avoid dehydration and consequent loss of elasticity with a consequent increase in the probability of fracture.

**FIGURE 1:**
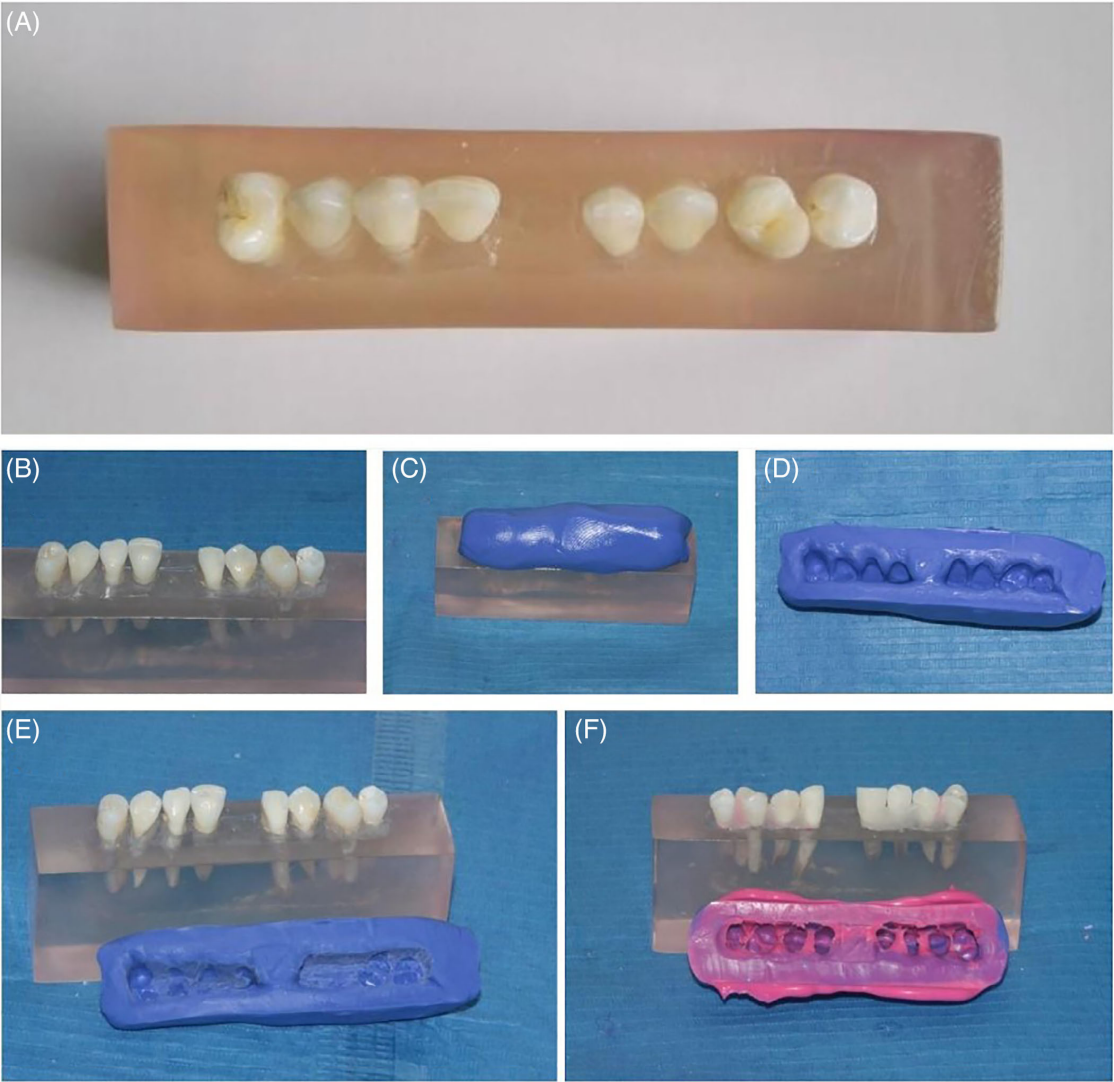
Model used to conduct the experiment. (A) “Occlusal” view. (B) “Lingual” view. (C) First phase of acquisition of a precision impression (positioning of the putty). (D) The putty impression. (E) Interdentations are removed from the putty prior to positioning the light body. (F) The final precision impression, after application and impression of the light body.

To carry out the tests, a rectangular wax mold (Tenatex Red, Kemdent) was made measuring 100 mm in length, 35 mm in width, and with a height of 25 mm. A transparent methyl methacrylate resin (Orthojet, Lang) was poured into this mold and carefully mixed in order to avoid the incorporation of air bubbles. This allowed the development of a base to be used to accommodate the selected teeth (Figure 1A,B). To allow proper polymerization, the resin was placed inside a polymerization machine (at a temperature of 30°C and at a pressure of 6 .08 × 10^5^ Pa) until complete hardening was achieved.

Once polymerization was complete, the resin block was modeled using a bur mounted on a special handpiece to allow the cropping up of the cemento–enamel junction. The teeth were then placed inside the resin block in line, with the crowns emerging from the resin, and were arranged in anatomical order leaving the space related to the missing central incisor; the contact points were reproduced and the residual spaces were filled with transparent fluid methyl methacrylate (Figure 1A,B). The in‐line positioning of the test teeth was chosen to allow the mechanical properties of the system to be studied.

The next phase of the test involved acquisition of a precision impression of the individual teeth on which to have indirect bonding prepared by the laboratory (Figure 1C–F). This phase was carried out by preparing a support for the impression material; the material of choice was a polyvinylsiloxane (Hydrorise, Zhermack) and the impression was subsequently sent to the specialized laboratory, Incognito Lab. The laboratory returned a set of customized attachments inserted in an indirect bonding device associated with a series of wires of different materials and shapes.

At this point, the individual teeth were sandblasted using a sandblaster with aluminum oxide of 50 µm, and then etched with 32% orthophosphoric acid for 30 s, followed by washing and drying for 30 s each. Test brackets (Customized Incognito lingual brackets with slot size 0.018″ × 0.025″) were attached to the teeth by indirect bonding using the specific tray sent by the laboratory and an orthodontic cement (RelyX Unicem 2 Automix; 3M  Unitek). After complete polymerization, the tray was removed, leaving the individual attachments visible, and a stainless‐steel (SS) wire of 0.018″ × 0.025″, supplied by the laboratory, was inserted and tied to the brackets.

The next step involved drowning the crowns of the individual teeth in a block of soft wax in order to make their position integral with their mutual relationship and anatomical order before changing their support. Then, using a dental bur, the model was segmented and each tooth was arranged on the line provided by the SS wire inserted in the slots of the brackets; the roots were then conveniently positioned to ensure the full passivity of the linear wire, and at this point resin was poured into the spaces left free from the drill.

### Tested wires

The Incognito laboratory sent the following seven types of wire for testing: 0.016″ × 0.022″ and 0.018″ × 0.025″ nickel–titanium (NiTi), 0.016″ × 0.024″ and 0.018″ × 0.025″ SS, and 0.017″ × 0.025″, 0.0182″ × 0.0182″ and 0.018″ × 0.025″ βIII titanium (βIIITi) (3M Unitek). Each wire was measured using a digital caliper to verify that the nominal dimensions corresponded with the real dimensions.

### Tested ligatures

**FIGURE 2:**
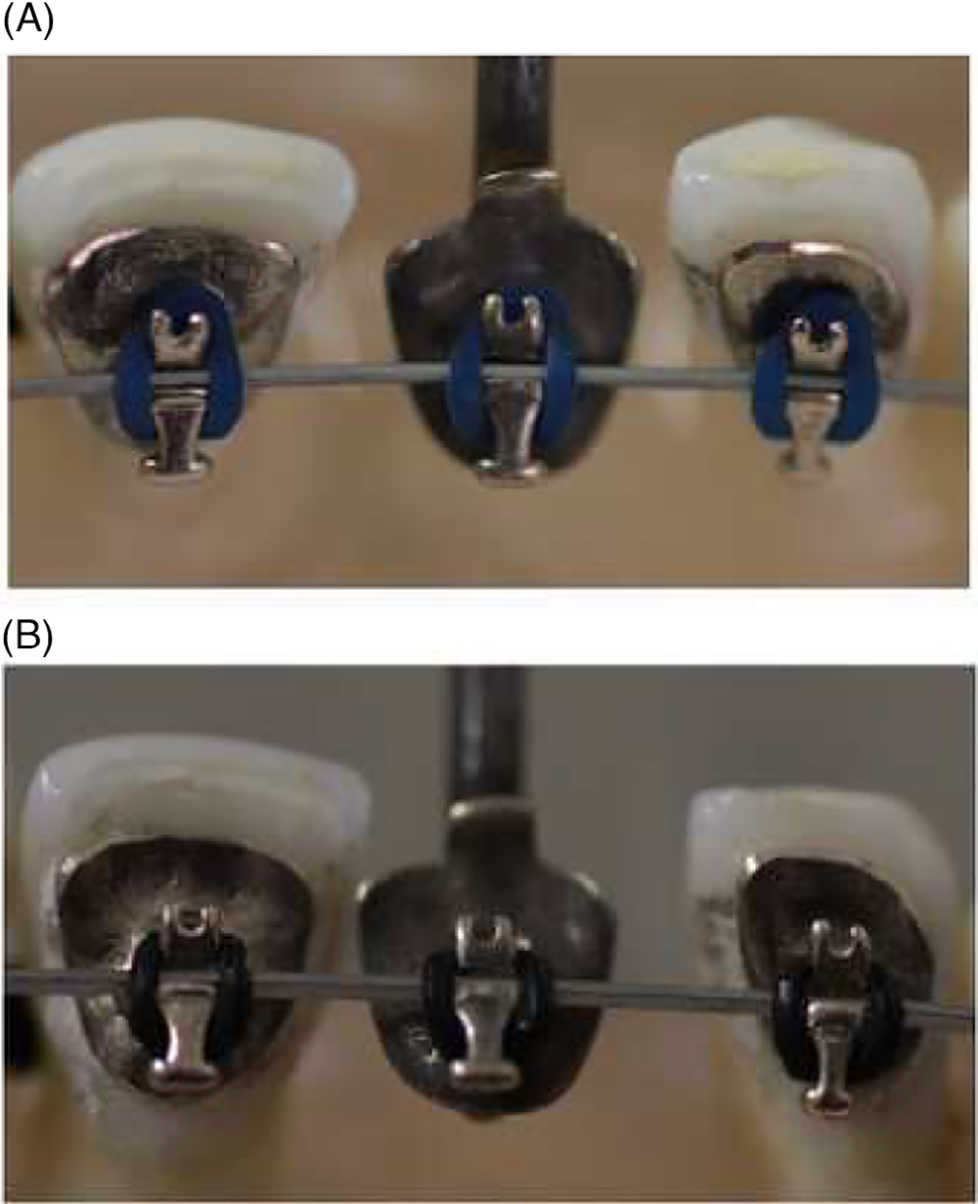
Macroscopic view of the typodont with different ligatures. (A) Alastik Lingual Ligatures. (B) Alastik Easy‐to‐tie.

Two types of ligatures were tested, namely Alastik Easy‐to‐tie and Alastik Lingual Ligatures (Alastik, 3M  Unitek) (Figure 2A,B).

### Testing machine

The bracket matching the missing incisor was tied to the wire and represented the test bracket on which the measurements were performed. An extension was laser‐welded to the bonding surface of the bracket to apply the forces to be tested. The extension was a large, round SS wire of 1.5 mm in diameter. At a distance of 10 mm, a bottleneck was shaped on the extension to create a detectable force application point for all of the tests. The extension was laser welded to the back of the bracket.

The Zwick/Roell Z0.5 testing machine (sensitivity, <1%; displacement sensitivity, 1 µm; full‐scale range, 500 N) was used to apply controlled traction and thrust forces to the system using forces from 0 to 0.5 N. The idealized cast was fixed to the Zwick/Roell Z0.5 machine with bolts and screws that engaged with the horizontal bar, so that the model was solidly fixed to the machine in order to create a system suitable for the application of forces.

**FIGURE 3:**
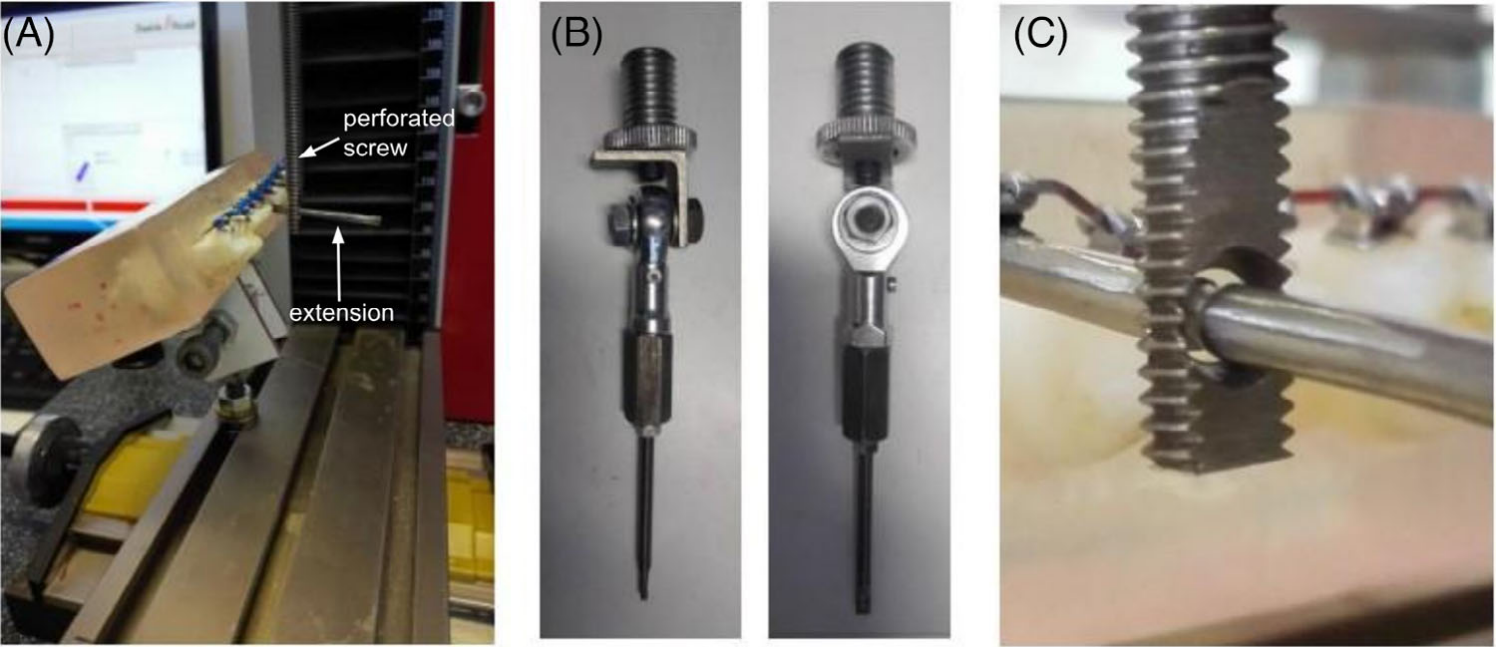
Details of the experimental set‐up. (A) Position of the model with respect to the traction—compression machine. The extension and the location of the perforated screw are pointed out. (B) Enarthrosis joint. (C) The perforated screw contacting the extension rod.

The model was positioned by placing the side with the largest area on the horizontal bar, so that the lingual side of each tooth was facing upwards, the vestibular side downwards, the coronal portion facing right, and the roots housed in the resin facing left (Figure 3A). The traction/compression system was hooked to the bottleneck extension of the test bracket via a rigid metallic perforated screw. The extension of the test bracket was placed into the screw hole (Figure 3A).

A connection similar to an enarthrosis joint was used between the screw and the machine in order to prevent decomposition of force in the horizontal component and to maintain constant vertical traction (Figure 3B). To eliminate play between the screw hole and the extension rod, a pretest adjustment was applied by moving the enarthrosis connection in such a position that the rod was in contact with the screw (Figure 3C).

**FIGURE 4:**
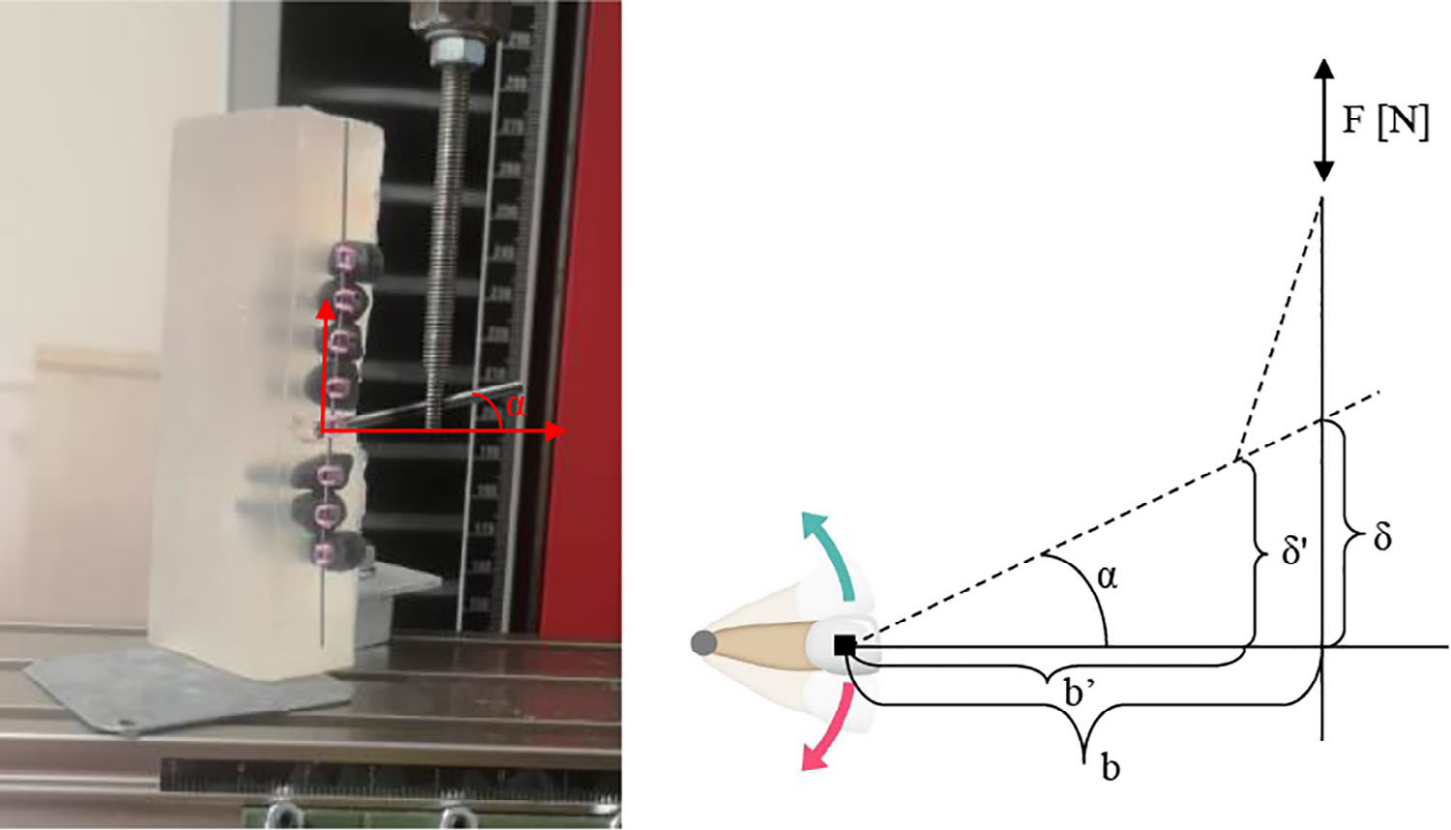
The experimental setting with a diagram of the applied force vector. The dashed lines represent the positions occupied by the extension of the test bracket and by the rigid metallic perforated screw connected to it when a constant vertical force is applied. The arm (b) of the force is changed during the movement (b’), as is the displacement (δ) induced by the extension (δ’). α, tipping angle.

The tipping moment (N∙mm) was calculated by multiplying the force by the length of the arm (i.e., the distance between the axis of rotation and the point of force application), which was fixed at 10 mm throughout the whole experiment. The tipping angle (°) was algebraically calculated because the machine (Zwick/Roell ZO.5) can measure the displacement induced by the extension. Hence, when the displacement (δ) and the arm (b) are known, the following holds (Figure 4):

sinα=δ/b

The tipping angle value (*α*) is a measure of the bending of the wire produced by the applied moment and was obtained by applying the arcsine function. When the angle *α* between the extension and the machine is changed, the arm b of the force changes (b′). After data collection, the actual arm was computed by correcting the initial length for the arm inclination cosine (Figure 4).

The bending angle of the center bracket at an applied moment from neutral to one side was used for the descriptive statistics and data analysis (a smaller angle represents better tip control of the bracket–wire system).

### Software

The testxpert ii (Zwick/Roell) software was used to record and analyze data and to illustrate the variation of the tipping moment into a Cartesian plane.

### Sample size

The sample size estimation resulted in six elastomeric ligatures per group being necessary to detect a difference of 3.5° in tip values (SD = 2.5°) assuming a significance level (alpha) of 0.05 and a power of 0.80. For each type of ligature, a sample of six ligatures was therefore tested for each wire.

### Statistical analysis

A blinded statistical analysis was performed. Tip angle values were tested for normality, and Levene's test was used to assess the homogeneity of variances. Differences among wires were evaluated using the Kruskal–Wallis rank sum test, and multiple post‐hoc comparisons were carried out using the Bonferroni method. Differences between ligatures were evaluated using the Mann–Whitney *U*‐test. Results are expressed as median and interquartile ranges, and differences with a value of *p* < 0.05 were selected as significant. Data were analyzed using the r v4.2.2 software environment [13]. The repeatability of wire measurements was evaluated using the intraclass correlation coefficient (ICC) and resulted in an ICC of >0.89 for all measurements.

## RESULTS

Descriptive statistics for tip angle absolute values are reported in Table 1. The angle calculated in the experimental set‐up represents the bending produced on the wire by the applied moment, rather than the tooth movement achieved. This angle is measured in the geometrical configuration that will produce the same moment once the wire is set into the slot. The smaller the angle, the greater the efficiency of the slot–wire–ligation system (because less bending produces the same moment). A statistically significant difference was found in angle values between ligatures when the complete set of data was tested (*p* < 0.001). Differences between ligatures were also found when testing each wire separately (Table 1). For every wire, Alastik Lingual Ligatures produced smaller angles than Alastik Easy‐to‐tie.

**TABLE 1:** Tip angle values stratified according to wire and ligature.

		Ligature		*Difference between ligature types (p*‐value)
Variable	Overall tip angle values for wire	Alastik Easy‐to‐tie	AlastiK lingual ligature	
Overall tip angle values for ligature		5.39 (2.70–9.65)	3.33 (1.56–5.55)	<0.001
Wire				
0.018″ × 0.025″ SS	4.09 (1.83–7.06)	6.03 (2.74–10.37)	3.03 (1.38–5.21)	<0.001
0.018″ × 0.025″ NiTi	3.41 (1.55–5.90)	4.15 (1.73–7.14)	2.85 (1.44–4.97)	<0.001
0.018″ × 0.0025″ βIIITi	2.75 (1.27–4.48)	3.82 (2.20–5.86)	1.87 (0.88–3.32)	<0.001
0.018″ × 0.018″ βIIITi	4.32 (2.18–6.99)	5.32 (2.88–8.74)	3.52 (1.70–5.59)	<0.001
0.017″ × 0.025″ βIIITi	4.59 (2.40–8.65)	5.30 (2.49–10.97)	4.10 (2.22–6.91)	<0.001
0.016″ × 0.024″ SS	5.06 (3.05–9.47)	7.29 (3.76–11.80)	3.50 (1.87–4.78)	<0.001
0.016″ × 0.022″ NiTi	6.65 (3.43–11.54)	8.37 (4.10–14.08)	5.56 (2.99–9.85)	<0.001
*Difference among wire types (p*‐value)	<0.001	<0.001	<0.001	

Abbreviations: βIIITi, βIII titanium; NiTi, nickel‐titanium; SS, stainless steel.

Values are given as median (interquartile range).

**FIGURE 5:**
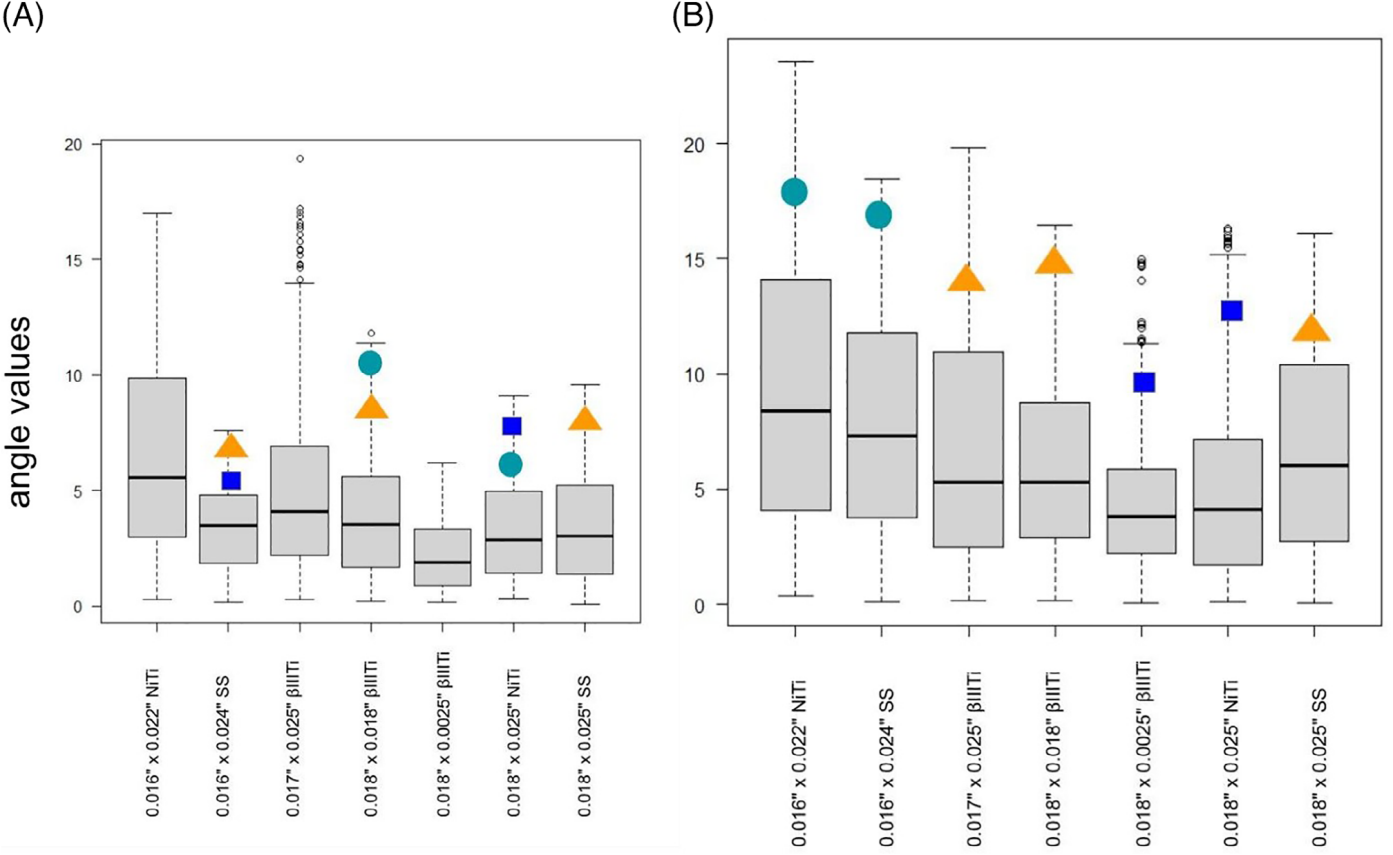
Comparisons between wires for each significant difference in angle values. Boxplots represent medians and interquartile ranges of angle values. Wires not showing any significant difference between them are labeled with the same symbol (a coloured square, triangle, or circle). Small grey circles represent outliers. (A) Comparison of AlastiK Lingual Ligatures. (B) Comparison of AlastiK Easy‐to‐tie. βIIITi, βIII titanium; NiTi, nickel‐titanium; SS, stainless steel.

A statistically significant difference was found among all wires with respect to angle values (*p* < 0.001, Table 1). Post‐hoc comparisons found significantly smaller angles for 0.018″ × 0.025″ NiTi and 0.018″ × 0.025″ βIIITi than for all the other wires (median values of the measured angles were 3.41° and 2.75°, respectively, *p* < 0.001 between them with the ligature Alastik Lingual Ligature; however, no significant difference was found with the ligature Alastik Easy‐to‐tie), while 0.016″ × 0.022″ NiTi showed the largest median angle value (6.65°). In other words, the best‐performing wires were able to express the same moment with bending 3°–4° smaller than the other wires. Pairwise comparisons between wires, when considering each ligature separately, are summarized in Figure 5A (AlastiK Lingual Ligatures) and Figure 5B (AlastiK Easy‐to‐tie).

## DISCUSSION

The findings of the present study show that wires that completely filled the slot in the bracket showed the best performance in terms of tip control, with an efficiency that appeared to be ligature‐related. The role played by the type of ligature was more relevant for wires that are undersized with respect to the slot dimensions; in particular, Alastik Lingual Ligatures offered better tip control (as indicated by their smaller angle values).

The wire material also contributed to tip expression; βIIITi performed better, that is, expressed lower angular deviation, than NiTi and SS wires of the same dimensions.

Proper three‐dimensional tooth control during orthodontic therapy is one of the key factors that needs to be managed by clinicians to obtain proper functional and esthetic results. This is true independently of the appliance used to reach that outcome. In the literature, the efficiency of tip control and the torque efficiency have been studied principally with buccal fixed appliances or with thermoformed aligners [14], and all the appliances showed a certain degree of imprecision or tolerance, or lack of expression, in the desired movement or final tooth position [15, 16].

Tip control using the Incognito lingual appliance may be problematic because of its ribbon‐wise slot, into which wires are inserted vertically. Inadequate tip control can lead to suboptimal esthetic outcomes. Given that vertical slots are used only in anterior teeth, they play a crucial role in finishing. The findings of this study suggest that using Alastik Lingual Ligatures in conjunction with smaller βIIITi archwires during the aligning and leveling phases may enhance tip control and improve overall treatment results.

Traditionally, different types of ligatures have been used to fill the slot completely and to improve tip control [17]. Recently, an experimental in vitro study on the tipping moment on anterior teeth produced by two types of lingual brackets with different ligatures and ligation methods took into consideration the stiffness of the system, that is, the tangent to the slope of the moment‐angulation curves. Particularly, the stiffness values for the Incognito brackets varied from 0.30 to 6.61 N∙mm/degree according to the various ligature types [18].

Moreover, in that experiment the Incognito bracket ligated with the Alastik Lingual Ligature showed an average of 0.78 N∙mm/degree stiffness (SD = 0.06) [18]. In the present study, the same bracket with the same ligature showed a stiffness varying from a median value of 0.92 (IQR = 0.71–1.14) to 2.69 (IQR = 2.27–3.61) N∙mm/degree (data not shown) depending on the wire. The stiffness value recorded by Reichardt et al. [18] was for a 0.025 × 0.018″ SS wire, which in the present study showed a median stiffness value of 1.73 (IQR = 1.42–2.26).

While the question on the importance of alternative ligation modes thus remains open, some of these differences could result from the use of different experimental settings; for example, the use of a typodont versus a single tooth. It is less questionable that a longer slot coupled with a horizontal insertion mode would be conducive for better results; in fact, both of these ideal settings would allow complete control. Further insight into bracket design efficiency would require repeating the experiment on self‐ligating and non‐customized brackets. Meanwhile, the present study suggests that even smaller size SS wires with a suitable ligature may provide a more appropriate tip control. The limitations of the present study are related to the evaluation of a single customized appliance; further studies are needed to assess the generalizability of the present findings to other appliances. Moreover, the in vitro model may not fully replicate the actual clinical conditions, considering the contribution of the periodontal ligament and bone structure and the presence of a liquid medium (saliva) to the mechanical response.

## AUTHOR CONTRIBUTIONS

**Conceptualization**: Marco Migliorati; Roberto Stradi; Sara Drago. **Methodology**: Alberto Lagazzo. **Software**: Sara Drago. **Validation**: Marco Migliorati; Roberto Stradi. **Formal analysis**: Sara Drago. **Investigation**: Luigi Rizzi. **Resources**: Marco Migliorati. **Data curation**: Sara Drago. **Writing—original draft** preparation: Sara Drago; Anna De Mari. **Writing—review** and editing: Marco Migliorati; Alberto Lagazzo; Maria Menini. **Visualization**: Sara Drago. **Supervision**: Maria Menini; Marco Migliorati. **Project administration**: Marco Migliorati. All authors have read and agreed to the published version of the manuscript.

## CONFLICT OF INTEREST STATEMENT

The authors declare no conflict of interest.

## FUNDING INFORMATION

This research did not receive any specific grant from funding agencies in public, commercial, or not‐for‐profit sectors.
